# A Novel Putative miRNA Target Enhancer Signal

**DOI:** 10.1371/journal.pone.0006473

**Published:** 2009-07-31

**Authors:** Thorsten Schmidt, Hans-Werner Mewes, Volker Stümpflen

**Affiliations:** 1 Helmholtz Zentrum München - German Research Center for Environmental Health, Institute of Bioinformatics and Systems Biology (MIPS), Neuherberg, Germany; 2 Chair for Genome-oriented Bioinformatics, Technische Universität München, Life and Food Science Center Weihenstephan, Freising-Weihenstephan, Germany; Tel Aviv University, Israel

## Abstract

It is known that miRNA target sites are very short and the effect of miRNA-target site interaction alone appears as being unspecific. Recent experiments suggest further context signals involved in miRNA target site recognition and regulation. Here, we present a novel GC-rich RNA motif downstream of experimentally supported miRNA target sites in human mRNAs with no similarity to previously reported functional motifs. We demonstrate that the novel motif can be found in at least one third of all transcripts regulated by miRNAs. Furthermore, we show that motif occurrence and the frequency of miRNA target sites as well as the stability of their duplex structures correlate. The finding, that the novel motif is significantly associated with miRNA target sites, suggests a functional role of the motif in miRNA target site biology. Beyond, the novel motif has the impact to improve prediction of miRNA target sites significantly.

## Introduction

MicroRNAs (miRNAs) are short (about 22 residues long) RNA fragments that play a crucial role in almost all cellular processes in animals as well as in plants [1], [2]. They influence protein expression by integrating into an active RISC complex capable to bind at a specific site to the target messenger RNA (mRNA) [3] thereupon inducing either cleavage of the identified target mRNA or stopping the translational process [3].

Currently, the identification of the miRNA target sites relies predominately on the complementarity of the miRNA and the mRNA target site sequence which is required for the binding of the miRNA. However, the shortness of the miRNAs yields to a very high number of feasible complementary target sites. For example, for about 20,000 human genes more than 800,000 target sites are predicted (miRBase target repository version 5 [4]). Beyond, it was shown that a perfect miRNA:mRNA duplex is not required for target site recognition [5]. Indeed, a perfect complementary between miRNA and target site was even shown to be entirely unable to confer regulation [6].

Up-to-date, numerous algorithms exit that attempt to identify potential miRNA target sites including TargetScan [7], [8], miRanda [9], PicTar [10], rna22 [11], Pita [12], and NBmiRTar [13]. In addition to miRNA and target site sequence complementarity, these tools differ by the way they utilize phylogenic conservation, RNA structural information, compositional features of the seed regions, and energy-based considerations. However, the overlap between target sites predicted by different algorithms is still rather limited [14]. Moreover, in contrast to the abundance of predicted miRNA target sites, only very a small fraction could be validated to be functional [15], [16].

Recent works indicate an increasing importance of the context of the actual miRNA target sites to enable regulation. For example, it was shown that in *C.elegans* the let-7 miRNA requires two unique target sites along with a linker region [17] to down-regulate the LIN-41 gene. Another example is the *C.elegans* lys-6 miRNA. Its target UTR of the gene COG-1 has also two miRNA target sites and additionally two further functional regions in close proximity of the target sites [6]. Besides, a PUF-type protein was recently shown to be involved in the regulation of a miRNA-controlled 3′UTR [18] and the DND-1 protein was shown to prohibit access of the miRNA to its target site by binding to the messenger RNA [19], [20].

Given the current level of knowledge, Didiano et al. [6] thus concluded that miRNA seed matches without taking context into account are not sufficient to predict regulating effects of miRNAs on their target transcripts. They propose that, analogously to transcription factors for gene regulation, further features beyond the target site are required for miRNA target control. However, although their work revealed two additional short regions in close proximity to the analyzed target site of the miRNA lys-6 in *C.elegans*, all of these features together showed to be not sufficient to confer regulation in UTRs of other worm genes. Moreover, all so far described features seemed to be hardly conserved in related genomes. Taken together, one may speculate whether the identified very short contextual features next to the miRNA lys-6 biding site are very specific with regard to regulation of the analyzed lys-6 miRNA.

In this work, we exceed previous analyses of single miRNAs and their target sites to a large-scale analysis of all experimentally supported human miRNA target sites. Instead of focusing on a single target site or miRNA, we took all described miRNAs and their target sites under consideration. Moreover, we extend the range of considered residues far beyond previous analyzes that were looking in sequence proximity of the target sites only.

As result, we present three novel major findings in this work. First, we present a novel widespread sequence motif in human mRNA transcripts which were shown to be regulated by miRNAs. We show that our motif can be found downstream in many of the experimentally supported human target sites. We also demonstrate that the novel motif shows no similarity to known regulatory motifs and has not been described as functional motif before. Second, we show that such regions associated with miRNA target recognition can be flexible located far away from the actual target site. This indicates that, similar to enhancer sites for genes and beyond current knowledge, close proximity to the controlled target site is not a necessity. Finally, we demonstrate that the novel motif influences the frequency of miRNA targets as well as the stability or the miRNA:mRNA binding duplex structure positively. This suggests that the novel motif represents a basic function in miRNA target biology.

In summary, we present a novel- and frequent GC-rich RNA motif that shows significant correlation with miRNA target sites in human. We show that the RNA motif is prone to improve the identification of actively and typically controlled miRNA targets. Beyond, we discuss that the novel type of motifs may represent a fundamental new discovery with general importance for miRNA target identification and control, comparable to transcription factors for the transcription process.

## Results

### Novel motif downstream of experimentally supported miRNA target sites

In this work we analyzed all available human messenger RNA with experimentally supported miRNA target sites obtained from the TarBase [21] repository. We confirmed that the majority of all validated miRNA target sites in 3′-UTR regions tend to reside nearer to the coding sequence (CDS) end than to the transcript end. For example, 50% of all validated miRNA target sites in human 3′-UTRs are at maximum 684 residues away from the CDS end, whereas the median space to the transcript end is 1127 residues (Figure S1).

**Figure 1:**
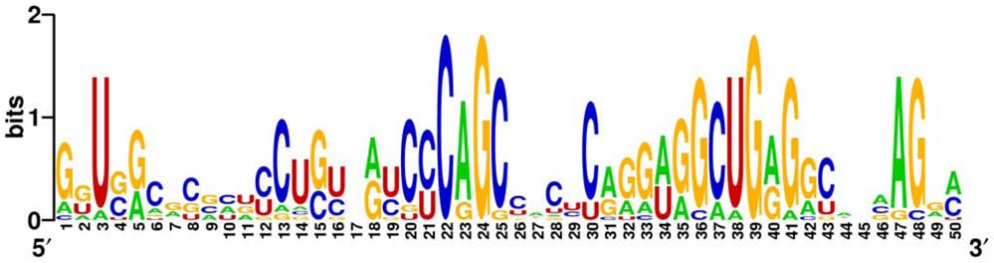
RNA motif. Detected novel miRNA target context sequence motif downstream of experimentally supported human miRNA target sites motif as sequence logo plot. The overall height of the stack indicates the sequence conservation at that position, while the height of symbols within the stack indicates the relative frequency of each nucleic acid at that position. Here the motif calculated from the non-redundant sequence set with maximum 65% allowed homology is shown. Noteworthy are the overall high GC content, the strong increase of cytosine nucleotides around the motif position 22 and a peak of guanine residues around motif positions 39.

In a first step, we analyzed the complete 3′-UTR sequence downstream of experimentally supported miRNA target sites with a spacer of 100 nucleotides to the target site as described in *Material and Methods* and shown in Figure S2. In the resulting non-redundant sequence set of human 3′-UTR sequences, we detected a novel sequence motif shown in **Figure 1**. This motif could be observed in 21% (12 of 57) of the sequences of the non-redundant sequence set. All hits are statistically significant with probabilities less than 10^-5^ to occur by chance. The probability of finding an equally well-conserved pattern in random sequences with the same nucleotide- and length distribution was estimated as less than 1.0^−46^ by the MEME algorithm [22], [23]. Furthermore, the motif is found identically no matter of the applied sequence homology filtering level. A detailed description of the motif along with a position weight matrix is given in File S1.

As second step, we evaluated whether the novel motif in the context of miRNA target sites has a similarity to a known functional motif. As described in the *Methods* section we used the T-Reg Comparator [24] tool to compare our motif to all 2203 known motifs included in the T-Reg web site search and to reveal potential (local) likeness. We found that the novel miRNA target context motif is different from any known transcription factor binding site stored in the databases TRANSFAC [25] and JASPER [26]. Moreover, the miRNA target context motif shows no similarity to the set of regulatory motifs in human promoters and 3′ UTRs that are conserved in several mammals [27]. This may indicate that the novel motif is organism specific. Furthermore, the PUF-type protein motifs [18], known ploy-A- [28], and splicing site-signals show no resemblance with the here reported motif and may be excluded as explanation. Beyond, we compared our motif with known microRNA binding sites utilizing the mirBase [4] data pool. All together, this suggests that the here reported motif is distinct from any known miRNA binding site and further known functional motifs in mRNAs.

### Novel motif occurrence and miRNA target frequency and duplex-stability correlates

The novel motif presented in this work was derived from 3′-UTR sequences downstream of experimentally supported miRNA target sites. However, the novel motif may be found together with miRNA target sites by chance. We therefore evaluated whether the occurrence of the novel motif has any impact of the frequency of miRNA target site occurrences and their prediction confidence.

As described in the *Methods* section, we splitted all 15,820 distinct human 3′-UTR sequences with miRNA target site predictions into transcripts with and without the novel motif. We found the novel motif in 33% (5,242 of 15,820) of the transcripts. The probability that a motif occurrence would be found by chance is less than 10^−4^ in all cases according to the MAST tool calculations [29].

**Figure 2:**
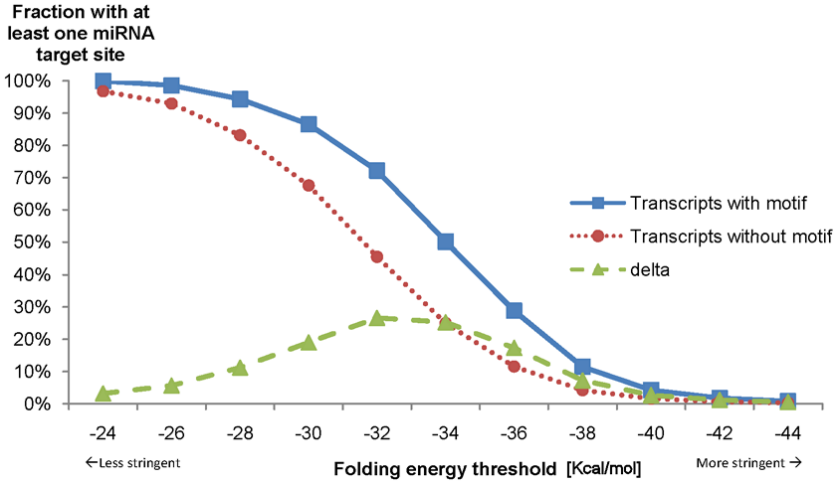
Percentage of distinct 3′-UTR transcripts having one or more miRNA target sites predicted. The folding energy indicates the stability of the RNA hetero duplex of the miRNA with the RNA binding site on the mRNA. Lower energy values indicate a stronger binding and are usually used as a parameter to filter miRNA target site predictions. Here, it can be seen that of the transcripts with the novel motif significantly more have at least one miRNA target site. The difference between transcripts-with-motif and transcripts-without-motif is shown as delta line. With increased stringent target site filtering, transcripts-with-motif show significantly more frequent at least one miRNA target site. Additionally, this indicates that the miRNA target site predictions in transcripts-with-motif have stronger miRNA:mRNA binding duplex structures on average.

First of all, we found that transcripts containing the novel motif have more miRNA target sites predicted in general. Additionally, miRNA target site predictions were filtered accordingly to their RNA duplex energy. The RNA energy of the miRNA:mRNA duplex indicates the stability of the miRNA binding. We observed that the higher miRNA target site density in transcripts containing the motif increases with more stringent filtering. If target sites with weak energy binding values are filtered out, the percentage of the transcripts-with-motifs that have at least one miRNA target gets up to 27% higher than transcripts-without-motif (**Figure 2**). This demonstrates that transcripts-with-motif have i) more predicted miRNA target sites and ii) that their predicted miRNA:mRNA duplex structures are more stable on average.

**Figure 3:**
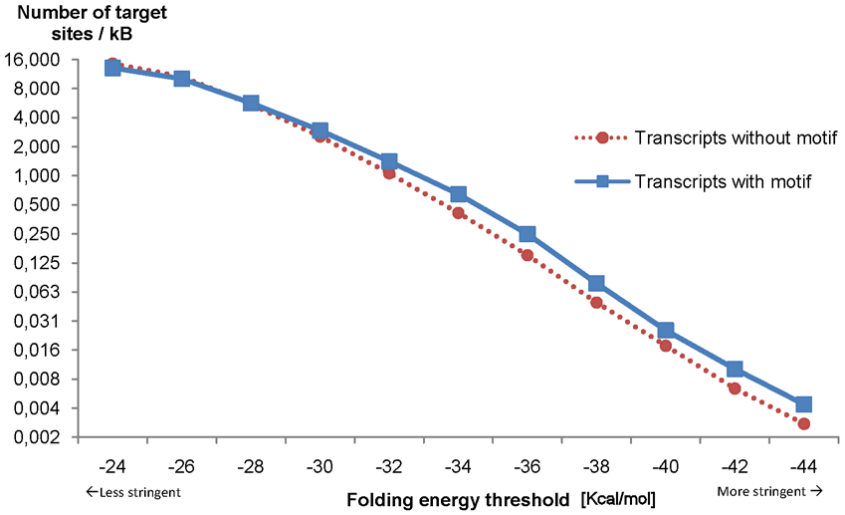
Number of predicted miRNA targets sites in transcripts with and without motifs. We conform that with increasing energy threshold the likelihood of predicted miRNA target sites decreases. Beyond, it can be seen that the amount of predicted miRNA target sites normalized by the sequence length is higher for transcripts-without-motif at first. However, regarding predictions that are supposed to be more stable (energy values get higher absolute values), more hits can be found in transcripts-with-motif.

We demonstrated that transcripts-with-motif show prevalence for more predicted miRNA target sites and that the modeled binding RNA duplex structures tend to be more stable. However, the absolute number of predicted miRNA targets may be influenced by the sequence lengths'. Indeed, the 3′-UTRs of transcripts-with-motif are with 1,875 nucleotides significantly longer than the ones of transcripts-without-motif being only 984 residues long on average. Therefore, we normalized the number of predicted miRNA target sites by the total sequence length of all transcripts of the respective set. As shown in **Figure 3**, it can be seen that for the least stringent energy threshold the frequency of a predicted miRNA target is greatest in transcripts-without-motif. However, as soon as the prediction specificity is increased by filtering out weak-energy predictions, the number of target sites per thousand nucleotides increases in the transcripts-with-motif. When all prediction with an energy value better or equal than −28 are considered, it can be seen that more miRNA target sites can be found in transcripts-with-motif independently of the sequence length. This underlines that the likelihood to find a miRNA target site in transcripts-with-motif is greater than in transcripts-without-motif as soon as the predictions are specific enough. This trend change may be explained by the assumed unspecific high number of false-positive miRNA target site predictions that can be partly filtered out by filtering by their RNA duplex energy values [11], [14], [30]. This indicates that the here observed trend will become even more clearly visible with improved data sets. It furthermore shows that transcripts-with-motif have more and more stable miRNA target sites independently of the sequence length.

## Discussion

In this work, we presented a novel- and frequent RNA motif that shows significant correlation with predicted miRNA target sites in human. We found that the occurrence of the novel motif increases the number of miRNA target sites as well as the stability of the binding RNA duplex structure. This suggests that the novel motif, which we derived from downstream sequences of experimentally supported miRNA target sites, is functionally connected with the miRNA target site biology. We suggest that the novel motif may act as miRNA target site enhancer similar to enhancers or transcription factors of genes.

We would like to point out that the novel motif is found in one third of the transcripts which were predicted to be controlled by miRNAs. This suggests that either the miRNA targets are highly over predicted or that further functional miRNA target enhancer motifs awaiting discovery. That fact, that we discovered only one motif in this work can be easily explained by the relatively small set of transcripts for which experimental data was available and which was used to derive a valid motif. Further motifs are likely to be discovered with more data at hand. Beyond, it remains to be investigated whether the enhancer-like character of our motif can be influenced by additional co-factors, and whether it is constant in all tissues and cellular contexts.

We further showed that the number of predicted miRNA target sites is higher in transcripts with the novel motif. We showed that this observation is independently of sequence length as long as miRNA target predictions are filtered by the hetero duplex folding energy. The miRNA:RNA duplex folding energy is one of the parameters widely used to improve and to evaluate the target prediction quality. Therefore, filtering with this criterion enables to decrease the number of false positive miRNA target predictions and leads to a clearer depiction of the function of the novel motif. However, given the inherent challenges of miRNA target prediction the duplex folding energy can improve of the prediction specificity only to a small degree. In consequence, even the most stringent filtering will contain a substantial amount of false predictions. This explains why the increase of the miRNA target frequency in transcripts with motif is relatively small compared to the frequency in the transcripts without the motif even when filtered by the energy parameter. Additionally, further motifs may act together to improve or suppress miRNA target sites. Under the assumption that further unknown factors are involved in miRNA target site regulation, the significant difference between transcripts with- and without motif points out the potential importance of the motif itself. Moreover, as we averaged organism-wide over all transcripts, the reported values may even increase as soon as more specific subsets will be analyzed.

In conclusion, the analysis of the context of experimentally validated human miRNA target sites revealed a novel non-coding motif on the mRNA level. This novel motif was found in about one third of the human 3′-UTRs of transcripts that are predicted to be regulated by miRNAs. The novel motif shows no similarity to previously reported functional domains on the mRNA level and can be found in far distance of the actual miRNA target site. Interestingly, the occurrence of the novel motif in a transcript's 3′UTR significantly increases the number of predicted miRNA targets independently of sequence length. Additionally, the predicted miRNA target sites show a stronger folding energy of the miRNA:mRNA hetero duplex if the novel motif was found in the transcript too. This indicates a more stable and trustworthy target recognition and processing in transcripts with the novel motif. Both, the derivation of the novel motif on experimentally supported data as well as the significant influence of the motif to miRNA target site frequency and stability suggests a functional relationship between the novel motif and miRNA target site processing. Analogously, to transcription factors and gene enhancers, the novel motif reported in this work may act as miRNA target site enhancer or silencer. While members of the Ago protein family play the central role in the RISC activated complex for miRNA:mRNA binding, it is known that during this process further proteins are recruited by RISC [31]. Because some of them (like GW128 or Rck) contain mRNA binding domains, one may speculate whether the motif acts as binding sites for them and therefore miRNA-RISC targeting is a cooperative effect. In any case, this would reveal a fundamental new biological layer of complexity. Independently of further experimental verifications, scanning the context of miRNA target sites for the novel motif has sound potential to improve all currently available miRNA target prediction methods.

## Materials and Methods

### Experimentally supported dataset of miRNA targets

MiRNA target site positions on the nucleotide level were extracted from the TarBase UCSC custom annotation tracks [21] as of August 2008. For human, 146 experimentally supported target sites were downloaded. The annotated human target sites range from 13 to 44 bases. On average, the human target sites are 24 nucleotides long. In the TarBase's UCSCS track, absolute genome coordinates of the miRNA target sites were provided for the genome assembly version hg17. Transcripts containing an annotated miRNA target site in their 3′ UTR were collected utilizing the corresponding UCSC known genes [32] dataset. To avoid ambiguous mRNA sequences, only validated RefSeq [33] transcripts (identifiers starting with “NM”) were used. This resulted in experimentally supported target site information for 118 distinct miRNAs on 147 transcripts (Supplementary Table S1). 95% (139 of 147) of these messenger RNAs have more than hundred residues downstream of the annotated miRNA target site on their 3′-UTR (Figure S2). This minimum length was chosen to exclude too short and non-informative sequences. As input for further analysis, all nucleotides downstream (i.e. towards the 3′ end of the mRNA) of the miRNA target site were taken. To avoid overrepresentation by similar and alternative (e.g. spliced) transcripts, all extracted sequences were made non-redundant on the sequence level. This was accomplished with the tool CD-HIT [34] with a maximum of 65% homology between representative sequences. This cut-off was the most stringent parameter setting applicable and ensures that the datasets show no substantial sequence similarity. As result, 57 representative non-redundant 3′UTR sequences downstream of validated miRNA targets remain.

### Motifs identification

The MEME [22], [23] standalone LINUX tool with default settings for DNA motifs was used for motif detection analogously as described before, for example see [35]. The MEME motif search was applied to the non-redundant downstream UTR sequence set which was derived from the TarBase data. Sequence plots were created with the WebLogo tool version 2.8 [36]. As input for the Logo creation all sequence blocks matching the motif were used.

### Motif comparison against previously reported signals

Indentified motifs were compared against each other using T-Reg Comparator [24]. The T-Reg tool provides a dissimilarity score of position weight matrices (PWMs) and considers local similarities as well as partly motif matches. We compared our motif against all known motifs from the transcription factor binding site databases TRANSFAC [25] and JASPER [26], and regulatory motifs in human promoters and 3′ UTRs that are conserved in several mammals [27] utilizing the T-Reg web interface [24]. Moreover, we compared our motif against known microRNA binding sites with the help of Amadeus' [37] supplementary compendium of target motifs derived from mirBase [4]. Beyond, the PUF-type protein motifs [18], known ploy-A- [28], and splicing site- signals were checked.

### Motif evaluation

A complete set of human 3′-UTR transcript sequences was downloaded from Ensembl release 50 database [38] utilizing the Biomart web tool [39]. Transcripts without 3′-UTR sequence, and entries that were not assigned unambiguously to a gene product on one of the chromosomes, were discarded. This resulted in 38,849 3′-UTR sequence entries. We will refer this set as complete human 3′-UTR set.

MiRNA target predictions for human 3′-UTR sequences were downloaded from the web site of the RNA22 miRNA target prediction tool [11] as of August 2008. RNA22 is founded on a pattern-based approach and does not rely on cross-species conservation. The latter feature ensures that species-specific sites can be detected. A further advantage of RNA22 is its sequence based pattern approach: the occurrence of further yet unknown motifs should not influence the RNA22 prediction quality of independent miRNA target site as long as no biological relationship is given. The precompiled RNA2 dataset contains predicted miRNA target sites on 15,820 distinct 3′-UTR transcripts of the complete human 3′-UTR set (matching accordingly to Ensembl's transcript IDs). Along other information, for each miRNA target site prediction, the folding energy of the predicted RNA hetero duplex (including linker contribution) is provided. We further ruled out a potential influence of the guanine- and cytosine- nucleotide frequencies (GC-content) of our sequences. As depicted in Figure S3, no significant correlation of the GC-content of the mRNA target sites with the folding energy could be noted.

All 3′-UTR sequences were checked whether they contain the novel motif using the MAST [29] motif search tool (the full transcript set including MAST results is available in Supplementary Table S2). Here the stand-alone implementation with default parameters and with the parameter *comp* to adjust P-values and E-values for each sequence by sequence's composition. Due to the strong signal of the motif occurrences, no differences in the number and positions of motif occurrences could be detected between both search runs. The set of 15,820 distinct 3′-UTR sequences with predicted miRNA targets was split into 5,242 distinct “transcripts-with-motif” and 10,578 distinct “transcripts without motifs” accordingly.

The “number of target sites per kilo bases (kB)” was calculated by summing up the number of predicted miRNA target sites and divide by the total sum of the sequence lengths' of the complete set respectively. This was done for the set of transcripts with and without motif. Additionally, filtering was applied to consider only miRNA target site predictions with a folding energy of the hetero duplex better than a given threshold. This measure indicates the likelihood of a miRNA target site within thousand nucleotides given a minimum folding stability criterion of the RNA hetero duplex.

## Supporting Information

Figure S1 Localization of experimentally described miRNA target sites on human transcripts. The box plots show the distribution of the space from the target site towards the CDS end (upstream of the target site) and to the transcript end (downstream of the target site). Here, all miRNA target sites in 3′-UTRs on validated human RefSeq transcripts are shown. The thick horizontal bars depict the median distance. Outliners above 5000 nucleotides are not plotted for clarity.
(0.00 MB TIF)

Figure S2 Schematic view of the analyzed mRNA transcript sequences with miRNA target sites. The complete remaining sequence downstream (i.e. in mRNA 3′ direction) of the target site were used with a spacer of 100 nucleotides.
(0.15 MB TIF)

Figure S3 GC content and miRNA: mRNA duplex structure free energy. Shown is the percentage of the nucleotides guanine and cytosine (GC-content) in the predicted miRNA target site sequences versus the minimum free folding energy of the modeled miRNA: mRNA duplex structure for all 283,273 predicted target sites. It can be seen that no significant correlation exists (Pearson correlation coefficient −0.28 with a p value<2.2–16). Due to the relatively small size of the miRNA target binding sites not all continuous GC percentage values are theoretically possible.
(0.09 MB TIF)

File S1 Motif details. Text file containing the motif along with position probability and weight matrix.
(0.02 MB TXT)

Table S1 Tarbase's human transcript data set. Excel sheet describing all used miRNA targets.
(0.09 MB XLS)

Table S2 Motif matches in all Ensembl transcripts. Excel sheet with all human transcripts in which 3′-UTR the motif was found along with hit details.
(3.69 MB XLS)
