# Supplementary figures and images for: A Novel Putative miRNA Target Enhancer Signal
Source: PLoS One. 2009 Jul 31;4(7):e6473. doi: 10.1371/journal.pone.0006473 (PMC2714067; doi:10.1371/journal.pone.0006473)

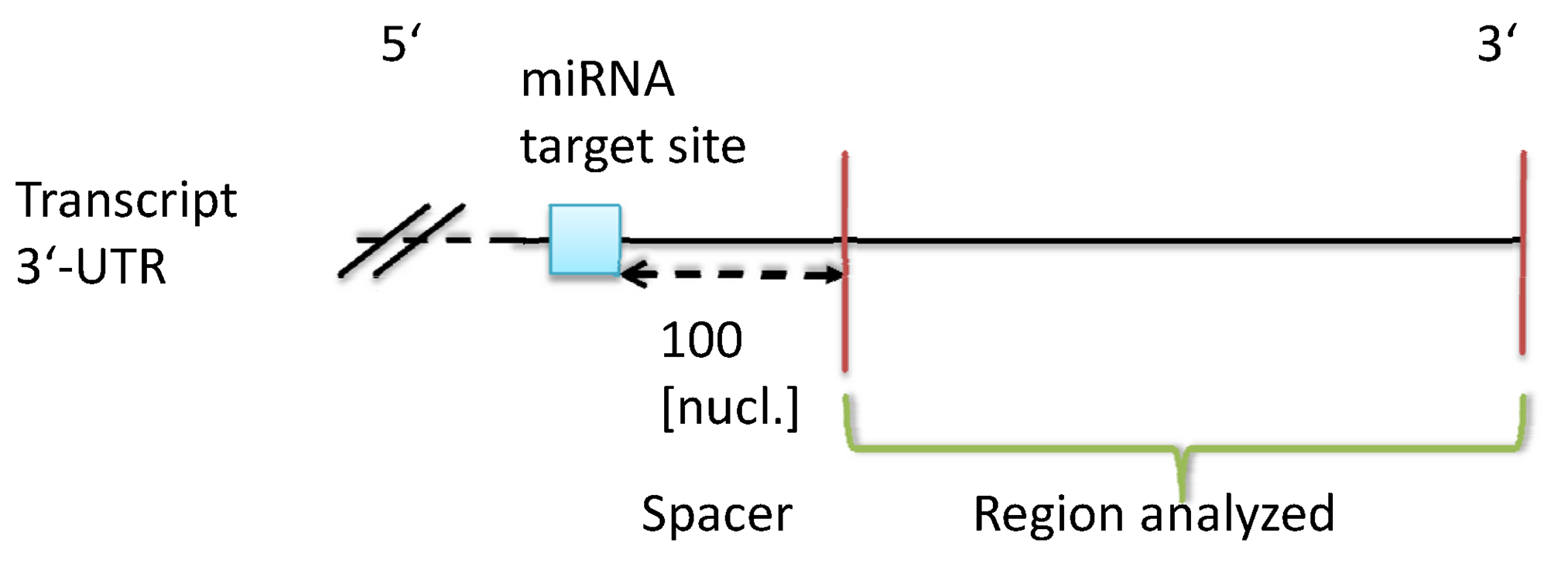

Supplement: Figure S2 — Schematic view of the analyzed mRNA transcript sequences with miRNA target sites. The complete remaining sequence downstream (i.e. in mRNA 3′ direction) of the target site were used with a spacer of 100 nucleotides. (0.15 MB TIF) [file pone.0006473.s002.tif]
